# Measuring Older Peoples’ Experiences of Person-Centred Coordinated Care: Experience and Methodological Reflections from Applying a Patient Reported Experience Measure in SUSTAIN

**DOI:** 10.5334/ijic.5504

**Published:** 2021-07-13

**Authors:** Jillian Reynolds, Erica Gadsby, Mieke Rijken, Annerieke Stoop, Mireia Espallargues, Helen M. Lloyd, James Close, Simone de Bruin

**Affiliations:** 1 Agency for Health Quality and Assessment of Catalonia (AQuAS), Department of Health of the Catalan Government, Barcelona, Spain; 2 Centre for Health Services Studies, University of Kent, Canterbury, UK; 3 Netherlands Institute for Health Services Research (Nivel), Utrecht, The Netherlands; 4 Department of Health and Social Management, University of Eastern Finland, Kuopio, Finland; 5 National Institute for Public Health and the Environment, Bilthoven, The Netherlands; 6 Amsterdam Public Health research institute, Amsterdam UMC – VU University Amsterdam, Amsterdam, the Netherlands; 7 Scientific Centre for Transformation in Care and Welfare (Tranzo), University of Tilburg, Tilburg, the Netherlands; 8 Agency for Health Quality and Assessment of Catalonia (AQuAS), Department of Health of the Catalan Government, Barcelona, Spain; 9 Spanish Health Services Research on Chronic Patients Network (REDISSEC), Barcelona, Spain; 10 School of Psychology, University of Plymouth, Plymouth, United Kingdom; 11 School of Medicine & Dentistry, University of Plymouth, Plymouth, United Kingdom; 12 National Institute for Public Health and the Environment, Centre for Nutrition Prevention and Health Services, Bilthoven, the Netherlands

**Keywords:** patient reported experience measures, integrated care, older people, person-centredness, care coordination, data quality, methods

## Abstract

**Introduction:**

While several evaluation studies on (cost-)effectiveness of integrated care have been conducted in recent years, more insight is deemed necessary into integrated care from the perspective of service users. In the context of a European project on integrated care for older people living at home (SUSTAIN), this paper shares the experience and methodological reflections from applying a Patient Reported Experience Measure (PREM) on person-centred coordinated care -the P3CEQ- among this population.

**Methods:**

A combination of quantitative and qualitative data and analysis methods was used to assess the usability and the quality of applying a PREM among older people presenting complex care needs, using the P3CEQ delivery in SUSTAIN as a case study. 228 service users completed the P3CEQ and nine SUSTAIN researchers participated in a consultation about their experience administering the questionnaire. P3CEQ scores were analysed quantitatively using principal component analysis and multilevel linear regression. P3CEQ open responses and researcher notes collected when administering the questionnaire were thematically analysed.

**Results:**

Service user inclusion was high and most P3CEQ items had low non-response rates. Quantitative analysis and researcher experience indicate the relevance of face-to-face administration for obtaining such an amount of data in this population group. The presence of a carer increased inclusion of more vulnerable respondents, such as the cognitively impaired, but posed a challenge in data interpretation. Although several P3CEQ items were generally understood as intended by questionnaire developers, the analysis of open responses highlights how questions can lead to diverging and sometimes narrow interpretations by respondents. Cognitive impairment and a higher educational attainment were associated with lower levels of perceived person-centredness of care.

**Conclusion:**

This study shows essential preconditions to meaningfully collect and analyse PREM data on older peoples’ experiences with integrated care: face-to-face administration away from care providers, collection of reasons for non-response and open comments providing nuances to answers, and multilevel modelling taking into account diversity in the target population. Several areas of improvement for future PREM use in this population have been identified: use of administration and coding guides, inclusion of clear and easy to understand definitions and examples illustrating what questions do and do not mean, measures of the expectations of person-centred coordinated care, and procedures ensuring sound ethical research. These methodological learnings can enhance future evaluation of integrated care from a service user perspective.

## Introduction

An increasing number of people with multiple health and social care needs live in their homes and communities until old age. Their complex care needs require multidisciplinary collaboration and coordination between care professionals from different organisations. Across Europe, numerous initiatives have been implemented to organise continuous and person-centred care for older people, often called integrated care [1,2,3,4,5,6]. Despite several evaluations, evidence for their (cost-)effectiveness is inconsistent [7,8,9,10]. This is partly due to diverse and often inappropriate outcome measures. These measures are often generic and health based (e.g. health status, physical functioning, quality of life) [8], thus failing to capture wellbeing, social participation and patient experience, arguably more appropriate for older people with complex needs [11,12,13]. Significant gaps in our ability to evaluate integrated care improvement from a service user’s perspective remain [14].

Care coordination and care tailored to peoples’ needs and preferences are principal characteristics of integrated care [15]. However, the multidimensionality and variety of values [16] attributed to integrated care hinders its measurement [17,18]. The concept of person-centred coordinated care (P3C) explicitly recognises the multidimensional nature of care experiences. P3C places an emphasis on understanding the relationship between individuals and their capabilities and resources, also acknowledging that care and support should strive to be responsive and coordinated across sectors, irrespective of organisational structures and configurations [19]. P3C is particularly relevant to assess care delivery for people who require continuous care, ensuring that patients are viewed as people in a care encounter, not just passive recipients [20].

Questionnaires can be useful tools to capture care experiences. There are a large number of tools exploring person-centred care [21,22]. However, there is a lack of Patient Reported Experience Measures (PREMs) that probe both coordination and person-centredness in a co-dependent model. Most existing PREMs (cf. Patient Assessment of Chronic illness Care (PACIC) [23], Picker Patient Experience Questionnaire (PPE-15) [24], Patient assessment of integrated elderly care (PAEIC) [25], IC-PREM-Home [26]), focus on these constructs separately. Moreover, the complexity of P3C can lead to ill-defined and abstract items that are difficult to understand or translate. Different individuals, for example, will have different understandings of ‘shared decision-making’ or ‘goal setting’. This difficulty is compounded for older people, who are more likely to suffer reduced cognitive function and sensory impairments, rendering questionnaire completion difficult. The testing of existing ‘user experience’ questionnaires with older people has sometimes found high respondent burden – with participation experienced as difficult, time consuming or emotionally stressful [27].

### Setting and aim

A PREM to measure Person-Centred Coordinated Care (P3CEQ) was developed between 2017–2019 in the United Kingdom [28,29] to address the growing priority of P3C for service users, carers, professionals and policymakers. Unlike pre-existing PREMs, the P3CEQ aims to jointly probe different aspects important in redesigning and integrating health and social care initiatives: person-centredness, care coordination, carer involvement and care planning [28]. The P3CEQ was used in a pan European project (SUSTAIN) between 2015–2019, aiming to generate evidence on how to improve integrated care for older people living at home with complex care needs [30]. SUSTAIN applied the P3CEQ as one of the instruments of a mixed-methods study design [31] to evaluate interventions in thirteen integrated care initiatives (details of SUSTAIN can be found elsewhere [32,33,34,35,36,37,38,39,40,41]).

The aim of this paper is to share the SUSTAIN experience and methodological reflections from applying the P3CEQ to explore care experiences of older people living at home with complex health and social care needs. The thirteen participating care initiatives were heterogeneous: from seven European countries, focused on different objectives and target groups, and providing different types of care and support services [33]. By sharing details of our data collection experiences, and analysing findings in relation to usability and quality of data, we hope to inform further development and use of PREMs to evaluate integrated care provided to (older) people with complex care needs.

The following research questions (RQ) guided our study:

RQ1. To what extent can older people with multi-morbidity and/or cognitive deterioration provide answers to a PREM exploring care coordination and person-centredness?

RQ2. What are the enabling and constraining factors for completing such a PREM instrument in this target group?

RQ3. Do service user characteristics or the administration mode have an impact on reported care experiences?

## Methods

### Study design: a case study on PREM use with older people

**Figure 1:**
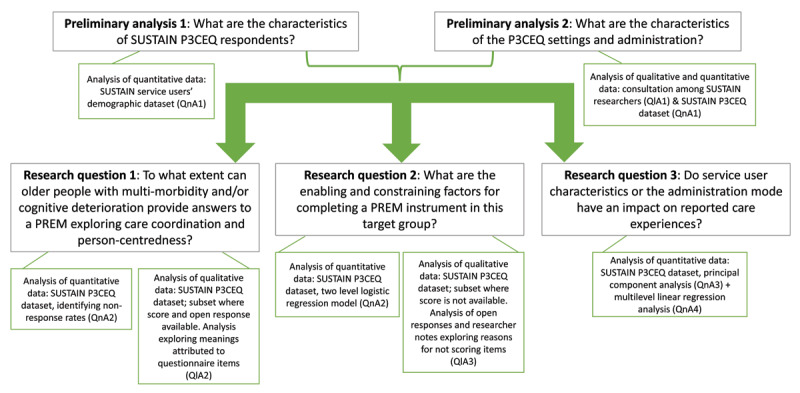
The SUSTAIN P3CEQ experience case study design: research questions, data and methods.

The SUSTAIN experience administering the P3CEQ is treated here as a case study of PREM use in older people with complex care needs. Our study combined quantitative and qualitative data and analysis methods to assess both the usability and the quality of PREM use (***Figure 1***).

### Measures and data collection

The P3CEQ is a valid and reliable 11-item measure of ‘person-centred coordinated care’ (***Table 3***) with strong face, construct and ecological validity [28]. In the P3CEQ validation study, a two factor measure was determined by principal component analysis: items 1–4 & 10 probe ‘person-centredness’ exclusively; items 6–7 probe ‘care coordination’, and items 5, 8 & 9 probe both constructs. Overall scalability was demonstrated by a Partial Credit Rasch analysis indicating good fit for each dimension. Furthermore, the P3CEQ showed longitudinal sensitivity to intervention change, which was confirmed by semi-structured interviews and ethnographic observation [29].

To enable its use in the SUSTAIN project, the P3CEQ was translated from the English-language version to six local languages (Catalan, Dutch, Estonian, German, Norwegian, Spanish) in collaboration with the original P3CEQ developers. This process was guided by principles of good practice for translation and cultural adaptation of patient reported outcome measures [42].

Subsequently, each integrated care initiative selected a convenience sample of service users following the minimum criteria defined by the SUSTAIN consortium (65+ years, living at home –unless temporarily admitted to a nursing home, with multiple health and social care needs as assessed by professional care teams, informed consent provided). Face-to-face administration was the preferred option to overcome possible limitations such as hearing, reading and writing difficulties. Researchers visited service users of ten initiatives at home, whereas three local research teams organised appointments at care provider premises. In exceptional cases, researchers delivered the P3CEQ as a postal survey or by phone (***Table 2***).

The P3CEQ includes tick boxes (for scoring) and open boxes (for comments). This study analysed both scores and comments, as well as any observational notes taken by researchers during/after P3CEQ completion. In addition, the study used sociodemographic and health data collected during the SUSTAIN project (sex, age group, completed education, living situation, self-reported chronic conditions and functional impairments).

For the purpose of this paper, nine researchers who delivered the P3CEQ to SUSTAIN service users were consulted to verify details concerning local administration methods using a self-complete questionnaire. We specifically enquired about interviewer procedures concerning repetition or rewording of questionnaire items to identify any differences across research teams. We also checked how SUSTAIN researchers coded reasons for not scoring items (i.e. using researcher notes, using the open text box of the corresponding question, etc.), to consistently incorporate this information into the dataset.

### Data analysis

Quantitative data analysis (QnA) consisted of four steps:

QnA1. Preliminary analysis described service user characteristics. From a list of eighteen individual health conditions, we created four categories of health characteristics that would be used in the multilevel analysis: hearing problems, visual problems, cognitive impairment and mental health problems (***Table 1***). Statistical descriptives were also calculated for P3CEQ context and administration mode variables.

QnA2. To address RQ1, we calculated the proportion of missing values for each P3CEQ item. Where this was higher than 10% [43,44], we addressed RQ2 by analysing whether *not* answering the item was related to service user characteristics or administration mode, using multilevel analysis. We estimated a two-level logistic regression model (level 1: integrated care initiatives, level 2: service users) predicting whether the service user had a missing value on that item (dependent variable). We estimated the total variance at the level of the integrated care initiatives with the variance at the level of the service users fixed at 1 (model 0). Then we estimated model 1, including a characteristic of the service users or administration mode (predictor variable) and estimated its fixed effect. This model was estimated for each characteristic separately.

QnA3. As a preliminary step to addressing RQ3, we conducted principal component analysis (with Varimax rotation) and compared the dimensionality of the P3CEQ with the two-dimensional structure found in the original validation study [29] and calculated Cronbach’s alpha. This was done to confirm we could construct two reliable scales (‘person-centredness’ and ‘care coordination’) for further analysis in QnA4.

QnA4. We conducted multilevel linear regression analysis to estimate the effect of the characteristics of service users and administration mode on the P3CEQ (scale) scores, thus addressing RQ3. As in QnA2, we estimated two two-level models: first a model 0, which included the two random coefficients (i.e. the variance components of each level) only, then model 1 in which we included a characteristic of the service users or administration mode as fixed coefficient (predictor variable). Model 1 was estimated for each characteristic separately, as the sample size did not allow multiple regression analysis. In QnA2 and QnA4, the regression coefficient and standard error, and the P-value based on the resulting Z-statistic are reported for the fixed effect of the characteristic included.

**Table 1:** Socio-demographic and health characteristics of the sample of service users (N = 228).


	N	M (SD)	n	%

**Socio-demographic characteristics**				

Sex: female	228		153	67.1

Age (in years):	228			

– 65–74 years			53	23.2

– 75–84 years			95	41.7

– 85 years and older			79	34.6

– Unknown			1	0.4

Education (completed):	228			

– No schooling/primary school			107	46.9

– Secondary school			48	21.1

– Advanced vocational training			48	21.1

– High professional/academic education			22	9.6

– Unknown			3	1.3

Living situation:	228			

– Living at home, alone			118	51.8

– Living at home, with spouse/partner			65	28.5

– Living at home, with family member(s)			20	8.8

– Living at home, with paid carer			2	0.9

– Assessed living/sheltered home			4	1.8

– Nursing or residential home for older persons			18	7.9

– Unknown			1	0.4

Marital status:	228			

– Married/cohabiting			78	34.2

– Divorced			26	11.4

– Widowed			105	46.1

– Single			16	7.0

– Unknown			2	0.9

Health related characteristics (self-reported)*				

Hearing problems:	226		92	40.7

Visual problems:	226		93	41.2

Cognitive impairments:	225		26	11.6

Mental health problems:	225		71	31.6

Number of chronic conditions**:	228	5.23 (2.47)		

– None			3	1.3

– One to three			60	26.3

– Four to six			101	44.3

– Seven or more			64	28.1

– Multi-morbid (2 or more chronic conditions***)			217	95.2


* The four categories of health characteristics were created from the list of eighteen individual health conditions collected with the demographic/health data sheet: hearing problems, visual problems, cognitive impairment (dementia including Alzheimers, loss of memory, traumatic brain injury, alone or in combination), and mental health problems (anxiety, panic disorders, depression, schizophrenia, alone or in combination). ** Variable based on a count of conditions indicated to be present. *** Multi-morbidity is calculated here as having two or more of the following conditions: hearing problems, problems with vision, dementia including Alzheimers, loss of memory, traumatic brain injury, anxiety – panic disorders, depression, breathing problems (asthma, chronic bronchitis, lung emphysema, or chronic obstructive pulmonary disease), cancer, diabetes, dizziness with falling, heart failure, stroke-cerebral haemorrhage, prostate symptoms, urine incontinence, broken hip, other broken bones, osteoarthritis, loss of bone tissue – osteoporosis, persistent back pain.

Analysis of qualitative data (QlA) consisted of three steps:

QlA1. Information obtained through the consultation with SUSTAIN researchers concerning administration mode and data coding was used to recode variables ensuring we applied consistent criteria in the use of each answer option in our final dataset.

QlA2. To provide a qualitative insight to RQ1, on the usability of this type of PREM by this target group, for each P3CEQ item we selected a subset of data where respondents had provided a score and an open response was available. We analysed these comments to identify how this subset of respondents interpreted the items and related them to their own experiences, providing details or examples of the meaning they attributed to each P3CEQ item.

QlA3. To address RQ2, for each P3CEQ item we selected the subset where a valid score was missing. We analysed open responses provided by service users as well as researcher notes to examine why the service user had not reported their experience using the corresponding scale. Explanations were categorised into possible reasons for missing scores using inductive coding.

## Results

### Characteristics of the study sample

SUSTAIN recruited service users in three iterations between 2016 and 2018. The majority completed the P3CEQ (93.4%; N = 228 of 244 total recruited service users [45]). Two thirds of the service users were women; three quarters aged 75 years or older (***Table 1***). About half of all service users did not complete any schooling or completed primary school only. Half of service users were living alone, whereas approximately 30% lived at home with their spouse or partner. Approximately 8% of service users were temporarily living at a home for older persons. The vast majority of service users suffered from multi-morbidity [46], presenting on average five chronic conditions. Osteoarthritis, persistent back pain and heart failure were reported most frequently. About 40% of the service users reported visual problems and a similar proportion reported hearing problems. Almost a third of service users reported mental health problems; 12% reported cognitive impairments.

### Characteristics of the P3CEQ administration

Sample sizes across the seven countries ranged from seven (Austria) to 61 (Germany) (***Table 2***). All but 11 service users completed the P3CEQ in a face-to-face interview. 72.8% responded to the P3CEQ at home; the others were interviewed at care provider premises (see methods). Seventeen percent of the service users completed the questionnaire in the presence of a family member/carer. For service users with cognitive impairment, this increased to 42.3%. Concerning the consultation among SUSTAIN researchers who administered the P3CEQ, all teams expressed that when needed they used additional agreed explanations/examples illustrating P3CEQ item meanings. Following SUSTAIN’s multi-method approach [32], a convenience sample of service users (N = 87) also participated in a qualitative interview during the same appointment.

**Table 2:** Country and P3CEQ administration characteristics.


	N	n	%

***Country***	228		

– Austria		7	3.1

– Estonia		52	22.8

– Germany		61	26.8

– Netherlands		13	5.7

– Norway		40	17.5

– Spain (Catalonia)		32	14.0

– United Kingdom		23	10.1

***P3CEQ administration characteristics***			

Mode of administering:	228		

– Face to face		217	95.2

– By telephone		3	1.3

– By mail		8	3.5

Place of administration	228		

– At home (includes temporary nursing home)		166	72.8

– At care provider premises		62	27.2

In presence of a carer:	228	39	17.1

Service users with cognitive impairment: In presence of a carer	26	11	42.3

In combination with qualitative interview:	228		

– No		141	61.8

– Qualitative interview before P3CEQ		33	14.5

– Qualitative interview after P3CEQ		54	23.7


### RQ1: To what extent can older people with multi-morbidity and/or cognitive deterioration provide answers to a PREM exploring care coordination and person-centredness

#### Quantitative analysis

Among our sample, missing answers per P3CEQ item were low at between 2.2% (for Q6 *Person(s) in charge of coordinating care*) and 18.9% (for Q11b *close ones involved in decisions as much as wanted*). Besides Q11b, only one other item had more than 10% missing values: Q5 *care joined up in a way that works* (12.3% missing values).

#### Insight: qualitative analysis of meanings attributed to ‘care coordination’ and ‘person-centredness’ construct items

The face-to-face administration of the P3CEQ provided a unique opportunity to explore understandings of different questionnaire items by older people, forming a *de facto* cognitive interview similar to those used during development processes of most PREMs. Although SUSTAIN researchers did not systematically collect understandings of all P3CEQ items for the complete sample, the subset of open responses provides insight into meanings this target group attributed to the questions.

Open responses were provided by 15–35% of respondents depending on the P3CEQ item: Q7 *care planning* obtained the least open responses (N = 34) and Q6 *professional coordinating care* obtained the most (N = 81). Open responses were coded as follows: ‘confirming’ (respondent recalls details or identifies an occasion that confirms a positive experience); ‘disputing’ (respondent recalls details or identifies an occasion indicating a less than positive experience); ‘neither’ (open response is not directly relevant to the question/neither confirms nor disputes a positive experience); and ‘both’ (respondent specifically recalls having both positive and negative experiences concerning the question).

Almost half of the open responses provided confirmed or explained a positive experience in relation to the item; a quarter reported less than positive experiences and fifteen percent were comments that neither confirmed nor disputed a positive experience. There were some particularities of P3CEQ items. For instance, for Q10 *confidence to self-manage health*, half the open responses reported a less than positive experience and frequently related this to the lack of personal autonomy.

***Table 3*** presents the most frequent concepts or meanings associated to each P3CEQ item, as identified in QlA2. Although not generalizable to the whole sample, some items were understood practically the same by most SUSTAIN respondents (e.g. Q4 *repeating information*, Q10 *confidence to self-manage health*), and in line with the meaning intended by P3CEQ developers, while other items were understood in a variety of ways, and not always coinciding with the intended meaning. Some concepts were narrowly understood: Q1 *discussing what’s important with care professionals* and Q2 *being involved in decisions* were frequently interpreted as having rather basic interactions with care professionals (e.g. being told what to do, being given advice); Q5 *care joined up in a way that works* was often related to how care professionals treat the respondents; and Q9 *receiving information to self-manage health* was answered on several occasions taking into account how much information was received in general.

**Table 3:** Meanings attributed to P3CEQ items in open responses of service users who scored the corresponding item. Ordered from more to less frequent.


	MOST FREQUENTMEANING/REACTION	OTHER FREQUENT MEANINGS/REACTIONS	OTHER MEANINGS/REACTIONS

Q1. Discuss what’s important with care professionals	Recalling (lack of) conversation with specific professional(s) or at a specific time (e.g. first visit), and/or (lack of) solution	Recalling basic interactions with care professionals (e.g. receiving advice, information, medication checks, being told what to do)	Reporting delegation of discussions to family member/friends Qualifying professionals (e.g. being happy with care team, trust, lack of empathy)

Q2. Involved in decisions	Recalling (lack of) being involved in a decision with specific professional(s) or at a specific time (e.g. first visit), and/or (lack of) solution	Recalling basic interactions with care professionals (e.g. receiving explanations, being informed, complaints being listened to, following routines)	Reporting cognitive deterioration as a factor to be taken into account when seeking decisions

Q3. Considered ‘whole person’	Recalling that care professionals(do/do not always or depending on the professional) treat them with e.g. caring attitude, compassion, respect, easy to understand language.		Referring to specific examples of how care professionals (did not) take whole situation into consideration (e.g. beyond clinical approach or criteria, beyond formal job duties) Recalling basic interactions (e.g. being asked ones opinion, receiving advice, being able to access electronic records)

Q4. Repeating information	Recalling how care professionals are (not) aware of conditions and/or can (not) access information (e.g. in the computer, written documentation, when care professionals change).		Recalling details that are not directly relevant to the question.

Q5. Care joined up in a way that works	Recalling how care professionals were (not) communicating, coordinating and aware of different parts of the care process	Qualifying professionals or professional care (e.g. well treated, useful, smooth) Referring to current health situation and (lack of) improved health outcomes	Referring to the existence of care plan that care professionals were following. Giving specific examples of how the coordinated care does (not) work for them (e.g. being visited at home, being visited by the same professional, long waiting lists, timetable of cleaners, coordination between primary and specialist professionals).

Q6. (Single) professional coordinating care	Confirming there is (not) a specific professional or professionals who coordinate care. (e.g the GP, the nurse, the GP and the nurse, one for social and one for health)	Identifying a family/friend as the person they were referring to as in charge of coordinating their care.	

Q7. Care planning (overall)	Describing the actions professionals and him/herself were applying as (not) part of a plan		Referring to medication plans or clinical records Referring to different needs that they consider (un)attended

Q8. Support to self-manage	Explaining whether care provided meets their needs		Referring to specific examples when advice or instrumental aid to enhance self-management were (not) being provided Qualifying professionals or professional care (e.g. well treated, helped when needed)

Q9. Information to self-manage	Recalling examples when (un)useful information for self-management was(not) received (e.g nutritional advice, medication adherence advice, overwhelming advice)	Refers to oneself or close persons as self-seeking information for self-management. Refers to receiving information in general.	

Q10. Confidence to self-manage	Refers to level of autonomy (physical, cognitive) as explaining level of confidence	Recalls examples of support (not) received and how that impacts level of confidence	

Q11a. Wants close ones involved	Identifies the person(s) to be involved. Identifies the person(s) to be involved, and specifies how or why		Explaining reasons for not wanting to involve others (e.g. self-capable, not wanting to be a burden, negative relation with family members)

Q11b. Close ones involved as much as wanted	Identifies persons or occasions when care team has/hasn’t involved as much as wanted.	Identifies the person whohas/hasn’t been involved	Refers to basic relations between care professionals and close ones (e.g. calling them, having them accompany service users to health consultations)


### RQ2: What are the enabling and constraining factors for completing a PREM instrument on care experiences in this target group?

#### Quantitative analysis

Analyses of the two items with non-response rates above 10% revealed that (non-)response could partly be explained by the integrated care initiative (intraclass coefficient of the null model was .26 (se .16) for Q5 and .36 (se .15) for Q11b). However, most variance in (non-)response to these items existed at the level of the individual service users.

The (fixed) effects of the service user characteristics (added in model 1) demonstrated some statistically significant findings (Supplementary file, Table 4):

1. Experiencing mental health problems was associated with less missing values for Q5 (coefficient –1.36, standard error 0.66, P = .04), indicating that service users with mental health problems were more likely to answer this item. In contrast, experiencing mental health problems increased the likelihood of not answering Q11b (coefficient 1.37, standard error 0.41, P = .001).
2. Service users with no schooling or primary schooling only were less likely to answer Q11b than service users who had completed secondary school (coefficient –2.24, standard error 0.83, P = .007).

The effects of other service user characteristics on (non)responding to Q5 or Q11b did not reach significance (Supplementary file, Table 4). For the other P3CEQ items the number of non-responders was too low to conduct these analyses.

The multi-level analysis of model 1 also provided some insights in whether the administration mode of the P3CEQ enabled or constrained answering Q5 and Q11b. It was found that:

1. Administering the P3CEQ otherwise than face-to-face increased the likelihood of non-responses for Q5 (coefficient 1.95, standard error 0.93, P = .04).
2. The presence of a carer during the interview decreased the likelihood of Q11b remaining unanswered (coefficient –1.36, standard error 0.62, P = .03).

The administration of the P3CEQ in combination with a qualitative interview did not impact the response of Q5 and Q11b (Supplementary file, Table 4). Again, the number of non-responders on the other P3CEQ items was too low to conduct these analyses.

#### Qualitative analysis

Lack of understanding or applicability of questionnaire items can also represent constraining factors for data collection. We analysed the open responses and researcher notes for the subsets where scores had not been provided to explore the different reasons explaining missing scores of P3CEQ items. Inductive coding identified six categories (Supplementary file, Table 5).

The most prevalent reason for not scoring questionnaire items was lack of relevance. It was more frequent for Q5 *care being joined up in a way that works* (N = 14), and was usually related to the perceived low complexity of the care they received, e.g. ‘I currently only receive care from one service’. It was also the most frequent cited reason for not scoring Q1 *discussing what’s important for your health and wellbeing with care professionals* (N = 12). In this case, three kinds of explanations were provided: 1) considering that ‘the care team knows best’; 2) considering that such discussions were pointless; or 3) relating the lack of relevance to the low frequency of visits with care teams. Lack of relevance was also the most frequent reason behind missing scores for Q2 *being involved in decisions* (N = 12) and was mostly related to the fact that the service user considered no decisions had been made (N = 9). Twelve cases considered Q9 *receiving useful information to self-manage* irrelevant, for instance because the respondent was highly dependent on others, or because respondents considered themselves self-sufficient, not needing such information from care teams.

### RQ3: Do service user characteristics or the administration mode have an impact on reported care experiences?

Our principal component analysis with Varimax rotation showed sufficient basis to calculate a person-centredness scale score similar to the result of the original validation study (by summing scores of Q1, Q2, Q3, Q4, Q5, Q8, Q9, and Q10), but not for calculating a care coordination scale score (Cronbach’s alpha .76 and .55 respectively) (Table 6, Supplementary file). Therefore, Q6 and Q7, which originally contributed with Q5, Q8 and Q9 to a care coordination scale, were analysed separately, in addition to Q11a and 11b, which were originally intended to be analysed separately.

Table 7 (Supplementary file) shows the mean scores of the service users on the P3CEQ person-centredness scale and Q7 and Q11b, as well as the percentages of service users answering ‘yes’ to Q6 and Q11a. In general, subgroups did not differ regarding their scores, with some exceptions. Service users aged 75 to 84 years were more positive about the care planning process (Q7) than the other age groups. Higher educated service users were less positive about the person-centredness of the care they received and the extent to which their carers were involved in decision-making about care (Q11b).

As to the effects of the various characteristics of service users and administration mode on the P3CEQ scores (***Tables 8*** and ***9***), the intraclass coefficient (ICC) of the null model for person-centredness was .24 (se .09), indicating that a substantial proportion of variation in service users’ scores related to the integrated care initiative. This might be explained by specific characteristics of the integrated care initiatives, but also by characteristics of health and social care systems where the initiatives had been implemented. Furthermore, a high level of education was related with experiencing care as less person-centred. In addition, experiencing cognitive problems related to experiencing less person-centred care.

**Table 8:** Fixed effects of characteristics of service users and administration mode on P3CEQ scale or item scores; results of two-level mixed-effect linear regression model (N = 13 integrated care initiatives, N = 183–225 service users); separate analyses for each characteristic.


	PERSON-CENTREDNESS (SCALE)				CARE PLANNING OVERALL (AVERAGE Q7a–d)				FAMILY/FRIENDS INVOLVED IN DECISION-MAKING AS MUCH AS WANTED (Q11b)			
		
	N	ESTIMATE	SE	P	N	ESTIMATE	SE	P	N	ESTIMATE	SE	P

**Fixed effect of service user characteristics**												

Gender: female (ref. male)	225	–0.20	0.60	.74	223	–0.08	0.15	.57	185	–0.15	0.15	.32

Age (ref. 65 to 74 years)	224				222				184			

– 75 to 84 years		–0.17	0.74	.82		0.34	0.18	.06		0.29	0.19	.12

– 85 years or older		0.71	0.74	.33		–0.11	0.18	.54		0.25	0.19	.19

Education (ref. no schooling or primary school)	222				220				183			

– secondary school		–0.81	0.79	.30		–0.21	0.18	.26		–0.33	0.19	.08

– advanced vocational training		–1.19	0.81	.14		–0.22	0.19	.25		**–0.46**	**0.22**	**.04**

– high professional/academic education		**–2.62**	**1.06**	**.01**		–0.25	0.25	.31		**–0.79**	**0.25**	**.002**

Hearing problems (ref. no)	223	–0.01	0.57	.99	221	–0.04	0.14	.79	184	0.13	0.14	.35

Visual problems (ref. no)	223	0.83	0.59	.16	221	–0.08	0.14	.56	184	–0.09	0.15	.54

Cognitive problems (ref. no)	222	**–2.43**	**0.86**	**.005**	220	–0.18	0.22	.40	183	–0.21	0.22	.35

Mental health problems (ref. no)	222	0.32	0.60	.60	220	0.09	0.15	.54	183	0.12	0.16	.45

**Fixed effect of administration characteristics**												

Mode of administering: other (ref. face-to-face)	225	2.82	1.61	.08	223	–0.29	0.37	.43	185	–0.26	0.40	.51

Carer: present (ref. not present)	225	–1.45	0.78	.07	223	–0.07	0.19	.73	185	0.17	0.20	.39

In combination with qualitative interview (ref. no)	225				223				185			

– interview before P3CEQ		–1.53	1.13	.17		–0.13	0.23	.57		0.25	0.28	.37

– interview after P3CEQ		–1.33	0.73	.07		–0.00	0.17	.98		–0.02	0.18	.90


**Table 9:** Fixed effects of characteristics of service users and administration mode on P3CEQ dichotomous item scores; results of two-level mixed-effect logistic regression model (N = 13 integrated care initiatives, N = 214–223 service users); separate analyses for each characteristic.


	(SINGLE) PROFESSIONAL COORDINATING CARE(Q6)				WANT FRIENDS/FAMILY INVOLVED IN DECISION-MAKING: YES (VS NO/DON’T KNOW) (Q11a)			
	
	N	ESTIMATE	SE	P	N	ESTIMATE	SE	P

**Fixed effect of service user characteristics**								

Gender: female (ref. male)	223	0.02	0.35	.96	217	–0.19	0.35	.58

Age (ref. 65 to 74 years)	222				216			

– 75 to 84 years		–0.48	0.45	.28		0.46	0.42	.28

– 85 years or older		–0.14	0.45	.75		0.81	0.42	.06

Education (ref. no schooling or primary school)	220				215			

– secondary school		0.26	0.49	.59		0.66	0.47	.16

– advanced vocational training		0.42	0.49	.39		0.32	0.46	.49

– high professional/academic education		0.91	0.65	.16		–0.32	0.58	.58

Hearing problems (ref. no)	221	–0.40	0.33	.23	215	0.67	0.34	.05

Visual problems (ref. no)	221	0.04	0.34	.91	215	–0.31	0.34	.37

Cognitive problems (ref. no)	220	–0.51	0.51	.32	214	0.76	0.61	.21

Mental health problems (ref. no)	220	0.13	0.35	.71	214	–0.37	0.34	.28

**Fixed effect of administration characteristics**								

Mode of administering: other (ref. face-to-face)	223	0.06	0.97	.95	217	–1.01	0.96	.29

Carer: present (ref. not present)	223	–0.03	0.45	.95	217	**2.12**	**0.66**	**.001**

In combination with qualitative interview (ref. no)	223				217			

– interview before P3CEQ		0.35	0.63	.58		1.02	0.66	.12

– interview after P3CEQ		–0.51	0.44	.24		–0.10	0.41	.82


Regarding service users’ experiences with care planning (Q7), the ICC of the null model was .04 (se .03), which means that differences in service users’ experiences with care planning were not related to the integrated care initiative they received care from. Neither the service user characteristics nor the way the P3CEQ was administered impacted on service user’ care planning scores.

The extent to which service users experienced that their carers (family or friends) were involved in decision-making about their care as much as they wanted (Q11b) related to the integrated care initiative they received care from (ICC .22, se .09). In addition, a higher level of education related to less positive experiences in this respect.

Whether service users stated they had a (single) care coordinator (Q6) was substantially related with the integrated care initiative they received care from (ICC .34, se .12). Service user characteristics and the way the P3CEQ was administered did not influence their answers to this question (***Table 9***).

Finally, whether service users needed or wanted their carers involved in decision-making about their care (Q11a) also related to the integrated care initiative they received care from (ICC .21, se .10). Service user characteristics were not significantly related to their answers to this question. Service users who had their carer present during the P3CEQ interview were more likely to confirm that they wanted their carers involved in decision-making about their care.

## Discussion Section

### Main findings and implications

Older people’s experiences with integrated care can be collected using a PREM instrument such as the P3CEQ. The findings of this case study provide insight into what worked and what could be improved when delivering a PREM with older and vulnerable populations and using data to assess integrated care from a service user perspective.

Unlike previous studies [26], SUSTAIN obtained a high return rate applying a PREM to evaluate care received by older people, reducing inclusion bias dramatically. All except two items of the P3CEQ had low non-response rates. The only potential reason explaining non-response that appeared across most items was a perceived lack of relevance of questions. Nevertheless, this lack of applicability was marginal (occurring for 72 of the 2,736 total possible scores; 2,736 = 228 respondents per 12 P3CEQ items). When answering RQ1, we must bear in mind that the usability of a PREM instrument is not just about how much data can be collected (i.e. return and response rates), but also the quality of data. A key aspect here is whether respondents understand questions and use tick boxes as intended. Our qualitative analysis provides insight on the possible shortcomings of the data obtained: while several P3CEQ items were generally understood as intended (e.g. Q4 *repeating information*, Q10 *confidence to self-manage health*), the analysis of open responses indicates how questions can lead to diverging interpretations by respondents. This highlights the difficulty of capturing data related to complex concepts quantitatively (particularly from populations like that in SUSTAIN), and also the importance of mixed methods and use of qualitative approaches such as in-depth interviewing to develop a nuanced understanding. PREMs such as the P3CEQ could be enhanced by including simple definitions with real-life examples illustrating each concept. This would help avoid narrow or misled interpretations such as understanding ‘being involved in decisions’ or ‘having discussed what is important’ as basic exchanges with care professionals; that ‘care joined up in a way that works’ is referring to the way care professionals treat service users; or that ‘support for self-management’ enquires about the extent to which support meets ones’ needs in general. If unidentified, these misinterpretations may lead to inaccurate assessments of experiences with integrated care.

Analysis of the enabling and constraining factors for questionnaire completion (RQ2) points to a key factor in this target group: face-to-face delivery. SUSTAIN researchers agreed that the high inclusion and response rates would unlikely have been feasible if the questionnaire had not been delivered face-to-face with service-users, at their own pace. After piloting the P3CEQ in the translation and cultural adaption process, SUSTAIN researchers opted for face-to-face interviews in order to maximize both quantity and quality of data. In fact, most SUSTAIN researchers expressed that they found themselves delivering the P3CEQ almost as a semi-structured interview guideline, providing additional explanations or examples to illustrate the meaning of items and facilitate understanding. In order to ensure data quality, researchers (and research funders) should be prepared for such a delivery, allowing sufficient time for each respondent to answer the questionnaire and adopting a facilitating role when needed. In this sense, researcher notes and paraphrasing of open responses are particularly relevant for data interpretability.

The presence of a carer while the questionnaire was delivered was another enabling factor. It had a significant impact on the level of response in one of the two questions with a non-response rate higher than 10% (Q11b), and became a more or less explicit requirement in cases presenting cognitive impairment. The option of using the carer as a proxy respondent enabled SUSTAIN’s research to be inclusive and provide learnings on care experiences of this particular target group, with carers commonly invited to participate in a qualitative interview [36]. However, presence of a carer introduced the possibility of bias. For instance, service users who had their carer present during the P3CEQ were more likely to confirm that they wanted their carers involved in decision-making about their care. Due to the collinearity between ‘existence of a carer’, ‘carer being present during questionnaire delivery’ and ‘cognitive impairment’ (when this applies), from a quantitative perspective we cannot make much of this data, and from a qualitative point of view we can only speculate the extent to which responses represented the carer’s or the service user’s wishes.

Our third RQ, exploring if user characteristics or administration mode impact the perceived level of person-centred coordinated care, provides three additional findings (besides the above-mentioned impact of presence of a carer) that are of methodological relevance concerning PREM design and data analysis. First, from a statistical point of view, it was not possible to obtain one of the two constructs intended by the P3CEQ –care coordination. Our dimensionality analysis therefore indicates the need for further validation among older and vulnerable people.

Second, service users with cognitive problems experienced less person-centred care as measured by the P3CEQ. This is in line with previous literature finding that, even in interventions designed specifically for the cognitively impaired, aspects relevant for person-centredness, such as communication and collaboration between family members and care professionals, can be lacking [47]. In this sense, it has been argued that person-centredness needs to be more proactively promoted within interventions, and this can be achieved through the inclusion of important and enjoyable –or meaningful [48] activities, both from the service user and carer perspective [49].

Third, service users with higher levels of education were less positive about the person-centredness of the care they received and about the extent to which their carers were involved in decisions. This coincides with previous studies exploring determinants of health care satisfaction and/or experiences [50,51]. Education and age can be used as proxy measures of health expectations, given their correlation with satisfaction [52,53]. Previously it has been hypothesised that older people may be more accepting and more reluctant to pass negative judgements with respect to their health care [52]. Cohen found that dissatisfaction with aspects of hospital-based care decreased markedly with age, but called for further research in order to confirm the aforementioned hypothesis [53]. A later study on the Questionnaire for Patient Expectations of Health Care found that older age predicted higher met expectations of health care [54]. The interrelation between age, health status, education, expectations and care assessment is an ongoing research topic [i.e. 52,53,50,55,54], that highlights the need to apply multivariate analysis and multilevel modelling, adjusting scores for the characteristics of the population in order to avoid systematic misrepresentations in the assessment of care that assists particular patient groups [55]. Such is the case for integrated care, a main beneficiary of which are older people who require continuous support from a variety of care providers. It is important, however, to recognise the diversity amongst older people, avoiding stereotypes [56]. Collecting and analysing user characteristics (e.g. physical functioning or autonomy, educational attainment, attitudes [50]) in relation to reported experiences with integrated care may help surface issues that are particularly relevant for specific subgroups. It would also be particularly useful to develop an instrument to collect expectations concerning the specific dimensions of person-centred coordinated care (e.g. discussions with care professionals, decision-making, self-management of health), as to improve the interpretation and use of data collected with PREM such as the P3CEQ.

Finally, although open responses to P3CEQ items gathered by SUSTAIN did not provide rich narratives, we can use them as indications of the kind of reactions people had when they completed this PREM. Questions on ‘support for self-management’ and ‘confidence to self-manage health’ triggered reflection on functional or mental impairments that might be hard to accept, acknowledging dependence on others. Question 11, which defines the concept of ‘close ones’ and asks if such persons should be included in decisions on care options, implies asking respondents to consider if they do or do not have anybody they can consider close, and if so, state if they do or do not want them involved. It is important to consider how questions in a tool like the P3CEQ might prompt discomfort amongst respondents, and make sure plans for limiting and dealing with such situations are in place to ensure sound ethical research. This is particularly relevant in cases where carers accompany service users when replying a PREM that includes questions enquiring about service user/carer relationship.

### Limitations

SUSTAIN researchers delivered the P3CEQ tool almost in the form of a semi-structured interview providing additional explanations when needed, and this helped reduce non-scoring to acceptable levels. However, researchers may have had different understandings and criteria on how to code certain answers. A guide specifying administration and coding criteria would be highly recommended since individual researchers might deal differently with situations where, for example, a respondent indicates a score then provides evidence that is contradictory to that score. This is particularly relevant when carers support service users to complete the questionnaire, since this implies having two persons –interviewer and carer- between the question and the service user, increasing the chance of differing interpretations.

Open responses were only provided by 15–35% of respondents who scored a P3CEQ item, and therefore the findings concerning how they understood each item cannot be generalised to the whole sample. There may be a bias in researcher note-takings, perhaps tending to write down responses more that deviated from their understanding of the concept. Further work, such as cognitive interviews, among frail older service user groups to check understandings of questions, concepts and scores may be valuable.

Finally, this paper does not emphasise the perceived level of person-centredness and coordination of care of the older people participating in SUSTAIN and how this varies across European integrated care initiatives. Nor does it examine factors particularly relevant in each integrated care initiative to explain service user experiences. Such analyses would be of interest, but are not possible here due to the combination of relatively low sample sizes from each integrated care initiative (since SUSTAIN’s multiple embedded case study design was characterised by the integration of evidence collected through a variety of instruments in order to identify patterns [57]), and the heterogeneity of the types of care and target groups of the thirteen initiatives involved in SUSTAIN.

### Conclusion

This study shows essential preconditions to meaningfully collect and analyse PREM data on older peoples’ experiences with integrated care: face-to-face administration away from care providers, collection of reasons for non-response and open comments providing nuances to answers, and multilevel modelling taking into account diversity in the target population. Several areas of improvement for future PREM use in this population have been identified: use of administration and coding guides, inclusion of clear and easy to understand definitions and examples illustrating what questions do and do not mean, measures of the expectations of person-centred coordinated care, and procedures ensuring sound ethical research. These methodological learnings can enhance future evaluation of integrated care from a service user perspective.

## Additional File

The additional file for this article can be found as follows:

10.5334/ijic.5504.s1 Supplementary File. Tables 4 to 7.

## References

[1] European Innovation Partnership on Active and Healthy Ageing. Repository of Innovative Practices. [cited 2020 March 14]. Available from https://ec.europa.eu/eip/ageing/repository_en.html.

[2] Hébert R, Durand PJ, Dubuc N, Tourigny A. PRISMA: A new model of integrated service delivery for the frail older people in Canada. International Journal of Integrated Care, 2003; 3: e08. DOI: 10.5334/ijic.73 16896376 PMC1483944

[3] Hopman P, de Bruin SR, Forjaz MJ, Rodriguez- Blazquez C, Tonnara G, Lemmens LC, Onder G, Baan CA, Rijken M. Effectiveness of comprehensive care programs for patients with multiple chronic conditions or frailty: A systematic literature review. Health Policy, 2016; 120(7): 818–832. DOI: 10.1016/j.healthpol.2016.04.002 27114104

[4] Kodner DL, Kyriacou CK. Fully integrated care for frail elderly: Two American models. International Journal of Integrated Care, 2000; 1: e08. DOI: 10.5334/ijic.11 16902699 PMC1533997

[5] Noordman J, Van der Heide I, Hopman P, Schellevis F, Rijken M. Innovative health care approaches for patients with multi-morbidity in Europe. NIVEL; 2015. [cited 2020 March 14]. Available from: https://www.nivel.nl/sites/default/files/bestanden/Rapport-CHRODIS.pdf.

[6] Van der Heide I, Snoeijs S, Melchiorre MG, Quattrini S, Boerma W, Schellevis F, Rijken M. Innovating care for people with multiple chronic conditions in Europe: An overview; 2015. Utrecht: NIVEL. [cited 2020 March 14]. Available from: https://www.nivel.nl/sites/default/files/bestanden/Rapport-State-of-the-Art-ICARE4EU.pdf. DOI: 10.1093/eurpub/ckv168.036

[7] Hoogendijk EO. How effective is integrated care for community-dwelling frail older people? The case of the Netherlands. Age and Ageing, 2016; 45: 587–590. DOI: 10.1093/ageing/afw081 27146300

[8] Looman WM, Huijsman R, Fabbricotti IN. The (cost-)effectiveness of preventive, integrated care for community-dwelling frail older people: A systematic review. Health Soc Care Community, 2019 1; 27(1): 1–30. Epub 2018 Apr 17 DOI: 10.1111/hsc.12571 29667259 PMC7379491

[9] Liljas AEM, Brattström F, Burström B, Schön P, Agerholm J. Impact of Integrated Care on Patient-Related Outcomes Among Older People – A Systematic Review. International Journal of Integrated Care, 2019; 19(3): 6. DOI: 10.5334/ijic.4632 PMC6659761 31367205

[10] Rijken M, Lette M, Baan CA, de Bruin SR. Assigning a Prominent Role to “The Patient Experience” in Assessing the Quality of Integrated Care for Populations with Multiple Chronic Conditions. International Journal of Integrated Care, 2019; 19(3): 19. DOI: 10.5334/ijic.4656 31592248 PMC6764181

[11] van Hees SGM, van den Borne BHP, Menting J, Sattoe JNT. Patterns of social participation among older adults with disabilities and the relationship with well-being: A latent class analysis. Arch Gerontol Geriatr, 2020 Jan-Feb; 86: 103933. DOI: 10.1016/j.archger.2019.103933 31542633

[12] McDonald KM, Sundaram V, Bravata DM, et al. Care coordination. vol. 7. Technical Review, ed. Rockville, MD: Agency for Healthcare Research and Quality Publication; 2007.

[13] Doyle C, Lennox L, Bell D. A systematic review of evidence on the links between patient experience and clinical safety and effectiveness. BMJ Open, 2013; 3: e001570. DOI: 10.1136/bmjopen-2012-001570 PMC3549241 23293244

[14] Scobie, S. Are patients benefitting from better integrated care? QualityWatch blog; 17 1 2019. Nuffield Trust and Health Foundation. [cited 2020 March 14] Available from: www.nuffieldtrust.org.uk/news-item/are-patients-benefiting-from-better-integrated-care.

[15] Goodwin N. Understanding Integrated Care. International Journal of Integrated Care, 2016; 16(4): 6. DOI: 10.5334/ijic.2530 PMC5354214 28316546

[16] Zonneveld N, Driessen N, Stüssgen RAJ, Minkman MMN. Values of Integrated Care: A Systematic Review. International Journal of Integrated Care, 2018 11 15; 18(4): 9. DOI: 10.5334/ijic.4172 PMC6251066 30498405

[17] King J, Gibbons E, Graham C, Walsh J. Developing measures of people’s self-reported experiences of integrated care. 2013. Picker Institute Europe and University of Oxford. [cited 2020 December 14] Available from: https://www.picker.org/wp-content/uploads/2014/10/Developing-measures-of-IC-report_final_SMALL.pdf.

[18] Lyngso AM, Godtfredsen NS, Host D, Frolich A. Instruments to assess integrated care: A systematic review. International Journal of Integrated Care, 2014; 14: None. DOI: 10.5334/ijic.1184 PMC4203116 25337064

[19] Lloyd H, Close J, Wheat H, Horrell J, Kirkpatrick T, Byng R, Valderas J, Collins A, Witts L, Sugavanam TP. How to use metrics, Measures and insights to commission person centred coordinated care; 2016. NIHR CLAHRC & NHS National Institute for Health Research. [cited 2020 March 14] Available from: http://p3c.org.uk/P3C_CommissionersGuide.pdf.

[20] Lloyd H, Jenkinson C, Hadi M, et al. Patient reports of the outcomes of treatment: a structured review of approaches. Health Qual Life Outcomes, 2014; 12: 5. DOI: 10.1186/1477-7525-12-5 24422873 PMC3899626

[21] da Silva D. Helping measure person-centred care; 2014. The Health Foundation. [cited 2020 March 14] Available from: https://www.health.org.uk/publications/helping-measure-person-centred-care.

[22] Lloyd H, Wheat H, Horrell J, Sugavanam T, Fosh B, Valderas JM, Close J. Patient-Reported Measures for Person-Centered Coordinated Care: A Comparative Domain Map and Web-Based Compendium for Supporting Policy Development and Implementation. J Med Internet Res, 2018 2 14; 20(2): e54. DOI: 10.2196/jmir.7789 29444767 PMC5830608

[23] Improving chronic illness care. PACIC survey. [cited 2020 December 14]. Available from: http://www.improvingchroniccare.org/index.php?p=PACIC_survey&s=36.

[24] Jenkinson C, Coulter A, Bruster S. The Picker Patient Experience Questionnaire: development and validation using data from in-patient surveys in five countries. Int J Qual Health Care, 2002 10; 14(5): 353–8. DOI: 10.1093/intqhc/14.5.353 12389801

[25] Uittenbroek RJ, Reijneveld SA, Stewart RE, Spoorenberg SL, Kremer HP, Wynia K. Development and psychometric evaluation of a measure to evaluate the quality of integrated care: the Patient Assessment of Integrated Elderly Care. Health Expect, 2016 8; 19(4): 962–72. Epub 2015 Jul 31. DOI: 10.1111/hex.12391 26230633 PMC5042070

[26] Teale EA, Young JB. A Patient Reported Experience Measure (PREM) for use by older people in community services. Age and Ageing, 2015 7; 44(4): 667–72. Epub 2015 Feb 21. DOI: 10.1093/ageing/afv068 25712515

[27] Muntinga ME, Mokkink LB, Knol DL, Nijpels G, Jansen AP. Measurement properties of the Client-centered Care Questionnaire (CCCQ): factor structure, reliability and validity of a questionnaire to assess self-reported client-centeredness of home care services in a population of frail, older people. Quality of Life Research, 2014 9; 23(7): 2063–72. Epub 2014 Feb 28. DOI: 10.1007/s11136-014-0650-7 24578148

[28] Sugavanam T, Fosh B, Close J, Phil D, Byng R, Horrell J, Lloyd H. Co-designing a measure of person-centred coordinated care to capture the experience of the patient. Journal of Patient Experience, 2018; 5(3): 201–211. DOI: 10.1177/2374373517748642 30214927 PMC6134538

[29] Lloyd H, Fosh B, Whalley B, Byng R, Close J. Validation of the person-centred coordinated care experience questionnaire (P3CEQ). International Journal for Quality in Health Care, 2019 8 1; 31(7): 506–512. DOI: 10.1093/intqhc/mzy212 30508089 PMC6839368

[30] de Bruin SR, Billings J, Stoop A, Lette M, Ambugo EA, Gadsby E, Häusler C, Obermann K, Ahi GP, Reynolds J, Ruppe G, Tram N, Wistow G, Zonneveld N, Nijpels G, Baan C, SUSTAIN consortium. Different Contexts, Similar Challenges. SUSTAIN’s Experiences with Improving Integrated Care in Europe. International Journal of Integrated Care, 2020 6 26; 20(2): 17. DOI: 10.5334/ijic.5492 PMC7319084 32607104

[31] Yin R. Case study research design and methods. 5th Edition. Thousand Oaks, CA: Sage Publications; 2014.

[32] De Bruin SR, Stoop A, Billings J, Leichsenring K, Ruppe G, Tram N, et al. On behalf of the SUSTAIN consortium. The SUSTAIN project: a European study on improving integrated care for older people living at home. International Journal of Integrated Care, 2018; 18(1). DOI: 10.5334/ijic.3090 PMC5887072 29632456

[33] Stoop A, de Bruin SR, Wistow G, Billings J, Ruppe G, Leichsenring K, Obermann K, Baan CA, Nijpels G. Exploring improvement plans of fourteen European integrated care sites for older people with complex needs. Health Policy, 2019; 123(12): 1135–1154. DOI: 10.1016/j.healthpol.2019.09.009 31615623

[34] Ambugo E, Hoel V, Hagen T. Sustainable tailored integrated care for older people in Europe (SUSTAIN-project): lessons learned from improving integrated care in Norway. Oslo: University of Oslo, 2018. [cited 2020 March 14]. Available from: https://www.sustain-eu.org/wp-content/uploads/sites/4/2018/08/Country-report-Norway_20180801.pdf.

[35] Billings J, Gadsby E, MacInnes J. Sustainable tailored integrated care for older people in Europe (SUSTAIN-project): lessons learned from improving integrated care in the United Kingdom. Canterbury: University of Kent; 2018. [cited 2020 March 14]. Available from: https://www.sustain-eu.org/wp-content/uploads/sites/4/2018/08/Country-report-United-Kingdom_20180801.pdf.

[36] De Bruin SR, Lemmens L, Baan CA, Stoop A, Lette M, Boorsma M, …, Minkman M. Sustainable tailored integrated care for older people in Europe (SUSTAIN-project): lessons learned from improving integrated care in the Netherlands. Bilthoven/Amsterdam/Utrecht: National Institute for Public Health and the Environment (RIVM), VU University Medical Center, Vilans; 2018. [cited 2020 March 14]. Available from: https://www.sustain-eu.org/wp-content/uploads/sites/4/2018/08/Country-report-the-Netherlands_20180801.pdf.

[37] Häusler C, Ruppe G. Sustainable tailored integrated care for older people in Europe SUSTAIN project: lessons learned From improving integrated care in Austria. Vienna: Austrian Interdisciplinary Platform on Ageing/OEPIA; 2018. [cited 2020 March 14]. Available from: https://www.sustain-eu.org/wp-content/uploads/sites/4/2018/08/Country-report-Austria_20180801.pdf.

[38] Hoffman H, Kamann D, Drews J, Claußen J. Sustainable tailored integrated care for older people in Europe (SUSTAIN-project): lessons learned from improving integrated care in Germany. Hamburg: Stiftung Gesundheit Fördergemeinschaft e.V., KV RegioMed Zentrum Templin, Pflegewerk Berlin; 2018. [cited 2020 March 14]. Available from: https://www.sustain-eu.org/wp-content/uploads/sites/4/2018/08/Country-report-Germany_20180801.pdf.

[39] Reynolds J, Masana L, Cayuelas Mateu N, Espallargues Carreras M. Sustainable tailored integrated care for older people in Europe (SUSTAIN-project): lessons learned from improving integrated care in Catalonia (Spain). Barcelona: Agency for Health Quality and Assessment of Catalonia (AQuAS); 2018. [cited 2020 March 14]. Available from: https://www.sustain-eu.org/wp-content/uploads/sites/4/2018/08/Country-report-Spain_20180801.pdf.

[40] Rull M, Tambaum T, Vainre M, Paat Ahi G. Sustainable tailored integrated care for older people in Europe (SUSTAIN project): lessons learned from improving integrated care in Estonia. Tallinn: Praxis Centre for Policy Studies Foundation; 2018. [cited 2020 March 14]. Available from: https://www.sustain-eu.org/wp-content/uploads/sites/4/2018/08/Country-report-Estonia_20180801.pdf.

[41] Lette M, Stoop A, Gadsby E, Ambugo EA, Mateu NC, Reynolds J, Nijpels G, Baan C, de Bruin SR. Supporting Older People to Live Safely at Home – Findings from Thirteen Case Studies on Integrated Care Across Europe. International Journal of Integrated Care, 2020 10 7; 20(4): 1. DOI: 10.5334/ijic.5423 PMC7546110 33100937

[42] Wild D, Grove A, Martin M, Eremenco S, McElroy S, Verjee-Lorenz A, Erikson P. Principles of good practice for the translation and cultural adaptation process for patient‐reported outcomes (PRO) measures: report of the ISPOR Task Force for Translation and Cultural Adaptation. Value in health, 2005; 8(2): 94–104. DOI: 10.1111/j.1524-4733.2005.04054.x 15804318

[43] Schafer JL. Multiple imputation: a primer. Stat Methods in Med, 1999; 8(1): 3–15. DOI: 10.1191/096228099671525676 10347857

[44] Bennett DA. How can I deal with missing data in my study? Aust N Z J Public Health, 2001; 25(5): 464–469. DOI: 10.1111/j.1467-842X.2001.tb00294.x 11688629

[45] De Bruin SR, Stoop A, Baan C, Nijpels G, Billings J, on behalf of the SUSTAIN Consortium. Sustainable tailored integrated care for older people in Europe (SUSTAIN-project): lessons learned from improving integrated care in Europe. Bilthoven/Amsterdam/Canterbury: National Institute for Public Health and the Environment (RIVM), VU University Medical Center, University of Kent; 12 2018. [cited 2020 March 14]. Available from: https://www.sustain-eu.org/wp-content/uploads/sites/4/2019/01/SUSTAIN-overarching-policy-report_final-version.pdf.

[46] Xu X, Mishra GD, Jones M. Evidence on multimorbidity from definition to intervention: An overview of systematic reviews. Ageing Res Rev, 2017 8; 37: 53–68. Epub 2017 May 13. DOI: 10.1016/j.arr.2017.05.003 28511964

[47] Spencer K, Foster P, Whittamore KH, Goldberg SE, Harwood RH. Delivering dementia care differently–evaluating the differences and similarities between a specialist medical and mental health unit and standard acute care wards: a qualitative study of family carers’ perceptions of quality of care. BMJ Open, 2013; 3(12): e004198. Published 2013 Dec 20. DOI: 10.1136/bmjopen-2013-004198 PMC3884743 24362015

[48] Kielhofner G. The model of human occupation: Theory and application. Baltimore, MD: Lippincott Williams & Wilkins; 2002

[49] Lu YY, Ellis J, Yang Z, Weaver MT, Bakas T, Austrom MG, Haase JE. Satisfaction with a family-focused intervention for mild cognitive impairment dyads. Journal of nursing scholarship, 2016 7; 48(4): 334–44. DOI: 10.1111/jnu.12214 27121662 PMC4970320

[50] Bleich SN, Ozaltin E, Murray CK. How does satisfaction with the health-care system relate to patient experience? Bull World Health Organ, 2009; 87: 271–8. DOI: 10.2471/BLT.07.050401 19551235 PMC2672587

[51] Roder-DeWan S, Gage AD, Hirschhorn LR, Twum-Danso NAY, Liljestrand J, et al. Expectations of healthcare quality: A cross-sectional study of internet users in 12 low- and middle-income countries. PLOS Medicine, 2019; 16(8): e1002879. DOI: 10.1371/journal.pmed.1002879 31390364 PMC6685603

[52] Hall JA, Doman MC. Patient sociodemographic characteristics as predictors of satisfaction with medical care: a meta-analysis. Soc. Sci. Med., 1990; 30: 811. DOI: 10.1016/0277-9536(90)90205-7 2138357

[53] Cohen G. Age and health status in a patient satisfaction survey. Soc Sci Med., 1996; 42: 1085–93. DOI: 10.1016/0277-9536(95)00315-0 8730914

[54] Bowling A, Rowe G, Lambert N, Waddington M, Mahtani KR, Kenten C, Howe A, Francis SA. The measurement of patients’ expectations for health care: a review and psychometric testing of a measure of patients’ expectations. Health Technol Assess, 2012 7; 16(30): i–xii, 1–509. DOI: 10.3310/hta16300 22747798

[55] Salisbury C, Wallace M, Montgomery AA. Patients’ experience and satisfaction in primary care: Secondary analysis using multilevel modelling. BMJ, 2010; 341: c5004. DOI: 10.1136/bmj.c5004 20940212 PMC2954274

[56] George T, Toze M, Ray M, Clayton O. Will the real “Mrs Smith” please stand up: a critical examination of the role of vignettes in integrated service development and delivery. Journal of Integrated Care; 2020 Vol. ahead-of-print No. ahead-of-print. DOI: 10.1108/JICA-05-2020-0031

[57] Glasgow RE, Green LW, Taylor MV, Stange KC. An Evidence Integration Triangle for Aligning Science with Policy and Practice. American Journal of Preventive Medicine, 2012; 42(6): 646–654. DOI: 10.1016/j.amepre.2012.02.016 22608384 PMC4457385

