# Supplementary material for: Measuring Older Peoples’ Experiences of Person-Centred Coordinated Care: Experience and Methodological Reflections from Applying a Patient Reported Experience Measure in SUSTAIN
Source: Int J Integr Care. 2021 Jul 13;21(3):3. doi: 10.5334/ijic.5504 (PMC8284500; doi:10.5334/ijic.5504)
Supplement: Supplementary File. — Tables 4 to 7. [file ijic-21-3-5504-s1.pdf]

Supplementary file:

Table 4. Fixed effects of characteristics of service users and administration mode on not reporting of P3CEQ items 5 and 11b; results of two-level mixed-effect logistic regression model (N=13 integrated care initiatives, N=228 users); separate analyses for each characteristic

	**P3CEQ item 5 missing (vs valid)**				**P3CEQ item 11b missing (vs valid)**			
	N users	estimate	se	P	N users	estimate	se	P
**Fixed effect of service user characteristics**								
Gender: female (ref. male)	228	0.36	0.48	.45	228	0.05	0.40	.90
Age (ref. 65 to 74 years)	227				227			
- 75 to 84 years		1.43	0.81	.08		0.19	0.53	.72
- 85 years or older		1.46	0.81	.07		0.12	0.54	.82
Education (ref. no schooling or primary school)	225				225			
- secondary school		-0.58	0.64	.37		**-2.24**	**0.83**	**.007**
- advanced vocational training		-0.30	0.66	.65		-0.35	0.51	.49
- high professional/academic education		-0.56	0.83	.50		-1.58	0.87	.07
Hearing problems (ref. no)	226	0.24	0.44	.58	226	0.10	0.39	.80
Visual problems (ref. no)	226	0.41	0.46	.38	226	0.32	0.40	.43
Cognitive problems (ref. no)	225	-0.03	0.63	.96	225	0.24	0.67	.72
Mental health problems (ref. no)	225	**-1.36**	**0.66**	**.04**	225	**1.37**	**0.41**	**.001**
**Fixed effect of administration characteristics**								
Mode of administering: other (ref. face-to-face)	228	**1.95**	**0.93**	**.04**	228	1.72	1.17	.14
Carer: present (ref. not present)	228	-0.36	0.71	.61	228	**-1.36**	**0.62**	**.03**
In combination with qualitative interview (ref. no)	228				228			
- interview before P3CEQ		-0.75	0.93	.42		-0.57	0.69	.40
- interview after P3CEQ		-0.14	0.55	.80		-0.44	0.53	.41

Table 5. Reasons identified in open responses or researcher notes explaining missing scores

**Category & meaning**	**Prevalence**	**Items where category was most prevalent**
Category A- Question irrelevant/not applicable: the respondent finds the question irrelevant or not applicable to his/her situation	72	Q5 *Care joined up in a way that works* (N=14)<br><br>Q1 *Discussing what is important with care professionals* (N=12)<br><br>Q2 *Being involved in decisions* (N=12)<br><br>Q9 *Receiving useful information to self-manage* (N=12)
Category B- Not understanding the meaning: the respondent fails to understand the meaning of the question, despite researcher facilitation, and cannot score his/her experience. For instance: "I do not know what you are referring to by [CONCEPT]"	16	Q2 Being involved in decisions (N=3)
Category C- response was irrelevant: the researcher considered that the respondent failed to grasp the meaning of the question since his/her response was irrelevant to the question and illustrated a lack of understanding, despite further clarifications provided by the researcher	9	Q3 Considered as a whole person rather than a disease (N=2)<br><br>Q6 (Single) named contact coordinating care (N=2)
Category D- question understood but answer not known: open response or researcher notes indicate that the respondent understands the question but does not know the answer	29	Q1 Discussing what is important with care professionals (N=8)<br><br>Q3 Considered as a whole person rather than a disease (N=5)<br><br>Q11b Close ones involved as much as wanted in decisions (N=5)
Category E- question understood but prefers not to anwer: the respondent understands what is being asked, but prefers not to answer	1	Q10 Confidence self-managing health and well-being (N=1)
Category F- reason for not scoring the item is unknown: neither a score nor the reason behind-non scoring was entered onto the P3CEQ	88	Q11b Close ones involved as much as wanted in decisions (N=30)<br><br>Q11a Wants close ones involved (N=9)

Table 6. Principle component analysis (Varimax rotation) of P3CEQ items

		Component 1	Component 2
Q1	Discuss what's important with care professionals	.537	.412
Q2	Involved in decisions	.751	.178
Q3	Considered 'whole person'	.647	.096
Q4	Repeating information	.411	-.378
Q5	Care joined up in a way that works	.748	-.025
Q6	(Single) professional coordinating care	.104	.768
Q7	Care planning (overall)	.087	.633
Q8	Support to self-manage	.743	.021
Q9	Information to self-manage	.536	.167
Q10	Confidence to self-manage	.466	-.261

Table 7. P3CEQ scale and item scores, mean scores or percentages for the total group of service users and subgroups

	**Person-centredness** (scale, 8 items, score range 0-24)			**(Single) named contact coordinating care** (Q6)		**Care planning overall** (average Q7a-d, score range 0-3)			**Wish to involve friends/family in decision-making** (Q11a)		**Family/friends involved in decision-making as much as wanted** (Q11b, score range 0-3)		
	**N**	**M**	**SD**	**N**	**%**	**N**	**M**	**SD**	**N**	**%**	**N**	**M**	**SD**
**Total sample**	225	17.42	4.81	223	54.7	223	0.64	1.05	217	66.4	185	2.31	1.06
**Gender**	$F(1,223)=0.02$, $P=.89$			$Chi^2(1)=0.06$, $P=.81$		$F(1,221)=0.26$, $P=.61$			$Chi^2(1)=0.61$, $P=.43$		$F(1,183)=1.02$, $P=.31$		
- women	153	17.39	4.97	152	55.3	150	0.61	1.03	147	64.6	124	2.26	1.10
- men	72	17.49	4.48	71	53.5	73	0.69	1.09	70	70.0	61	2.43	0.97
**Age**	$F(2,221)=0.64$, $P=.53$			$Chi^2(2)=0.36$, $P=.83$		**$F(2,219)=4.50$, $P=.01$**			$Chi^2(2)=5.74$, $P=.06$		$F(2,181)=1.83$, $P=.16$		
- 65 – 74	53	16.75	5.01	52	53.8	53	0.54	0.95	53	52.8	44	2.05	1.22
- 75 – 84	92	17.67	4.78	92	53.3	90	0.89	1.18	88	69.3	75	2.41	1.01
- 85 and older	79	17.50	4.70	78	57.7	79	0.43	0.89	75	72.0	65	2.37	0.99
**Education**	**$F(3,218)=6.76$, $P<.001$**			$Chi^2(3)=2.34$, $P=.51$		$F(3,216)=1.27$, $P=.29$			$Chi^2(3)=4.33$, $P=.23$		**$F(3,179)=8.84$, $P<.001$**		
- no/primary school	106	18.65	3.94	104	57.7	103	0.78	1.15	104	67.3	82	2.70	0.75
- secondary school	47	17.30	4.81	47	55.3	48	0.53	1.02	47	76.6	46	2.22	0.99
- vocational training	48	16.29	5.38	48	45.8	48	0.48	0.84	43	62.8	35	1.91	1.27
- higher education	21	14.22	5.69	21	61.9	21	0.50	0.89	21	52.4	20	1.65	1.39
